# Electronic-Medical-Record-Driven Machine Learning Predictive Model for Hospital-Acquired Pressure Injuries: Development and External Validation

**DOI:** 10.3390/jcm14041175

**Published:** 2025-02-11

**Authors:** Kim-Anh-Nhi Nguyen, Dhavalkumar Patel, Masoud Edalati, Maria Sevillano, Prem Timsina, Robert Freeman, Matthew A. Levin, David L. Reich, Arash Kia

**Affiliations:** 1 Institute for Healthcare Delivery Science, Icahn School of Medicine at Mount Sinai, New York, NY 10029, USA; 2 Wound and Ostomy Care Service, The Mount Sinai Hospital, New York, NY 10029, USA; 3 Department of Anesthesiology, Perioperative and Pain Medicine, Icahn School of Medicine at Mount Sinai, New York, NY 10029, USA; 4 Department of Genetics and Genomic Sciences, Icahn School of Medicine at Mount Sinai, New York, NY 10029, USA; 5 Windreich Department of Artificial Intelligence and Human Health, Icahn School of Medicine at Mount Sinai, New York, NY 10029, USA

**Keywords:** hospital-acquired pressure injury, pressure ulcer, machine learning, predictive model, multi-center validation, external validation, electronic medical records, clinical decision support, wound care management, clinical decision support, automated EMR integration

## Abstract

**Background:** Hospital-acquired pressure injuries (HAPIs) affect approximately 2.5 million patients annually in the United States, leading to increased morbidity and healthcare costs. Current rule-based screening tools, such as the Braden Scale, lack sensitivity, highlighting the need for improved risk prediction methods. **Methods:** We developed and externally validated a machine learning model to predict HAPI risk using longitudinal electronic medical record (EMR) data. This study included adult inpatients (2018–2023) across five hospitals within a large health system. An automated pipeline was built for EMR data curation, labeling, and integration. The model employed XGBoost with recursive feature elimination to identify 35 optimal clinical variables and utilized time-series analysis for dynamic risk prediction. **Results:** Internal validation and multi-center external validation on 5510 hospitalizations demonstrated AUROC values of 0.83–0.85. The model outperformed the Braden Scale in sensitivity and F1-score and showed superior performance compared to previous predictive models. **Conclusions:** This is the first externally validated, cross-institutional HAPI prediction model using longitudinal EMR data and automated pipelines. The model demonstrates strong generalizability, scalability, and real-time applicability, offering a novel bioengineering approach to improve HAPI prevention, patient care, and clinical operations.

## 1. Introduction

Hospital-acquired pressure injuries (HAPIs) represent a prevalent and largely preventable complication within inpatient settings, with an estimated global prevalence of approximately 12% among hospitalized adult patients. Despite extensive efforts to enforce international guidelines and implement various risk assessment tools, such as the Braden and Norton scales, their incidence continues to significantly impact patient care and impose multi-billion-dollar costs on healthcare systems worldwide [1].

Since 2014, many published studies [2,3,4,5,6,7,8,9,10,11,12,13,14,15,16] have tried to use machine learning techniques to predict the risk of hospital-acquired pressure injuries. To contextualize the novelty of our work, we conducted a comprehensive state-of-the-art review of 37 published studies identified through a targeted PubMed search using the query: (“pressure injury”[Title] OR “pressure ulcer”[Title]) AND (“artificial intelligence”[Title] OR “machine learning”[Title] OR “deep learning”[Title] OR “predictive model”[Title]) between 1 January 2014 and 14 October 2024. Of these, 22 studies were excluded as they fell outside our study scope, focusing on pediatric or emergency department populations, reviewing existing literature, or aiming to diagnose or stage existing pressure injuries rather than predict risk. The remaining 15 studies were systematically evaluated against the following innovative criteria:

- Development of a predictive model for HAPI risk: This study develops and validates a machine learning model specifically to predict HAPI risk
- Use of raw EMR data: The model directly processes real-world, un-curated EMR data, without pre-standardization or alteration beyond the original clinical observations
- Inclusion of all inpatient service lines: The model predicts HAPI risk across the whole adult inpatient population, without restrictions to specific service lines, conditions, or age brackets (e.g., critical care units only)
- Integration of longitudinal time-series EMR variables: The model incorporates time-series data, i.e., each EMR variable is sampled from different timestamps throughout the patient’s hospitalization, rather than relying solely on static or aggregated observations
- End-to-end automated pipeline: The model is fully integrated into the EMR. It is an end-to-end pipeline that starts with obtaining the real-world EMR data in the raw format, performs transformations (normalization, encoding, null imputation, sampling, and time series), and generates a prediction score
- External multi-hospital validation: The model is externally validated on independent patient cohorts from hospitals not included in the development phase

**Figure 1:**
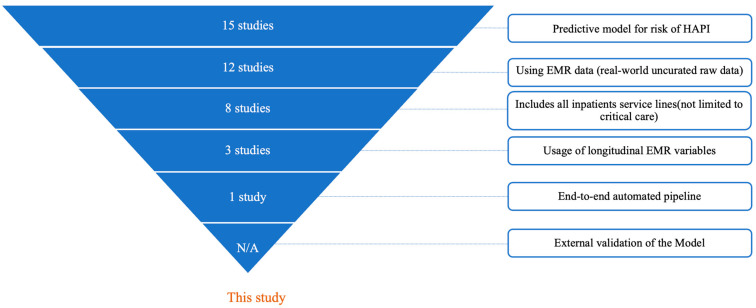
Funnel graph showing the number of similar published studies [2,3,4,5,6,7,8,9,10,11,12,13,14,15,16] by criteria of review.

Each study was evaluated against each criterion. Figure 1 is a funnel graph showing similar published studies by each of these criteria of review. No study satisfied all the criteria simultaneously except our study (Figure 1).

Indeed, the goal of this study is to develop a high-performing, interpretable HAPI risk prediction model that is fully automated from data extraction through prediction generation and has the potential to be seamlessly integrated into electronic health record (EHR) systems as an early warning system for nurses and clinicians.

Among the 15 reviewed studies [2,3,4,5,6,7,8,9,10,11,12,13,14,15,16], twelve [2,3,4,5,6,8,9,11,12,13,15,16] utilized raw clinical data directly extracted from a hospital-integrated system. One of the remaining studies [10] used ICD-10 diagnosis codes which are part of billing data. Two of the remaining studies [7,14] relied on the MIMIC dataset, which, while de-identified and multi-institutional, poses integration challenges for hospital EHR systems and limits applicability beyond critical care patients. Four studies [2,4,8,15] further constrained their scope by focusing exclusively on critical care units, where HAPI incidence rates range from 4.9% to 33%, compared to 0.52–4.4% in the other studies considering inpatient populations. This discrepancy in prevalence shifts the predictive problem toward a rare-event classification with high class imbalance, impacting model performance and generalizability.

Of the eight studies [3,5,6,9,11,12,13,16] incorporating all inpatient service lines, most employed traditional machine learning models such as support vector machines, Random Forest, XGBoost, or LSTMs, reporting AUROC values between 0.72 and 0.99 and sensitivities between 0.74 and 0.87. However, only three accounted for time-dependent changes in patient condition. Some relied on static or aggregated features, such as Song J. et al. [6] who averaged clinical variables over a fixed window or such as Song W et al. [3] who only used the most recent measurement of each clinical feature. Such approaches fail to capture the evolution of patient risk over time, limiting their operational feasibility in real-world hospital settings.

Three prior studies [9,11,12] incorporated longitudinal EMR variables, but only Dweekat OY et al. [9,12] proposed an end-to-end automated pipeline suitable for EHR integration. However, this study included the Braden Scale as a predictive feature. In contrast, our model benchmarks performance against the Braden Scale rather than relying on it, ensuring improved risk assessment accuracy.

The final key differentiator of our study is external validation. All 15 prior studies used internal validation, meaning their models were trained and validated within the same hospital system, potentially compromising generalizability. Our study evaluates model performance across four independent hospitals, demonstrating superior robustness and scalability for real-world implementation.

By integrating dynamic clinical variables into a time-series framework, leveraging an end-to-end automated EMR pipeline, and conducting extensive multi-hospital validation, our approach addresses critical gaps in existing research. This study introduces a scalable, clinically applicable solution for improving HAPI prevention and enhancing patient outcomes across diverse healthcare settings.

## 2. Materials and Methods

### 2.1. Study Setting, Population, and Data Sources

This study was undertaken at five Epic facilities in The Mount Sinai Health System (MSHS): The Mount Sinai Hospital (1139-bed academic hospital), Mount Sinai West (514 beds), Mount Sinai Morningside (489 beds), Mount Sinai Brooklyn (212 beds) and Mount Sinai Beth Israel (543 beds). Data were collected from two EHR platforms—Epic (Epic Systems, Verona WI) and Cerner (Cerner Corporation, North Kansas, MO, USA). This study adhered to the Transparent Reporting of a multivariable prediction model for individual prognosis or diagnosis statement [17]. All methods were performed in accordance with relevant guidelines and regulations provided by the Institutional Review Board (IRB), which granted a waiver of informed consent (IRB-18-00573-MODCR001).

In all the sections below, we are using the following abbreviated names for each facility: “Facility A” for The Mount Sinai Hospital, “Facility B” for Mount Sinai Morningside, “Facility C” for Mount Sinai Brooklyn, “Facility D” for Mount Sinai West, and “Facility E” for Mount Sinai Beth Israel.

Facility A was used for model training and retrospective internal validation. Facilities B, C, D, and E were used for retrospective external validation.

**Figure 2:**
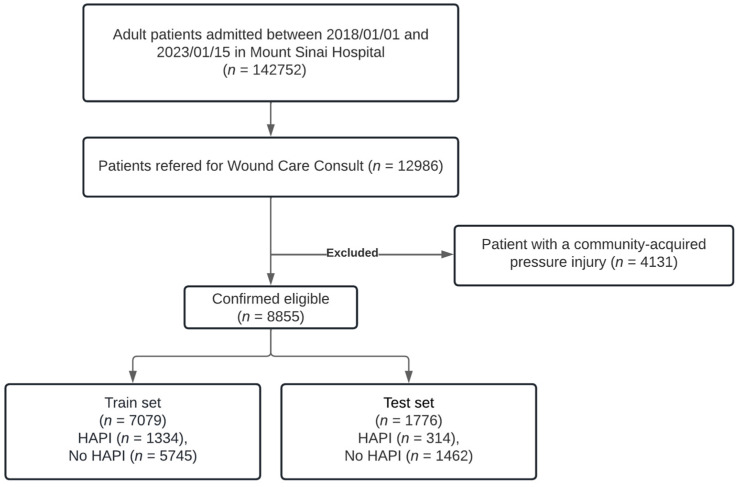
Patient flow and inclusion/exclusion criteria in the development cohort.

In the development cohort, used for model training, we included adults (age ≥ 18 years) admitted to Facility A from 1 January 2018 to 15 January 2023, who were referred for a wound care consult. Patients with a community-acquired pressure injury were excluded. Figure 2 shows the flow chart for inclusions and exclusions for the cohort.

### 2.2. Labeling Logic 

One significant challenge encountered in this study was the lack of structured, systematic, and reliable documentation of HAPIs across Mount Sinai Health System facilities. The primary and most accurate source for identifying HAPIs was wound care consultations performed by Wound Care Nurses (WOCNs), who are considered the clinical experts in this domain. The documentation of pressure injuries that were not validated by WOCNs was deemed unreliable. To address this, specific labeling logic was developed and validated with assistance from the lead WOCN. This validation process involved a comprehensive chart review of 197 hospitalizations from MSHS, during which the lead WOCN reviewed, corrected, and confirmed the accuracy of the HAPI labels. A target labeling accuracy of 95% was set as the threshold for finalizing the labeling logic.

### 2.3. Label and Clinical Feature Sampling Strategy

The outcome was HAPIs classified as stage 1 to 4, deep-tissue injury, or unstageable.

All inpatient hospitalizations were labeled based on the following logic.

- If the HAPI happened within the inpatient hospital length of stay, the label was positive, and the label time stamp was the HAPI timestamp.
- Otherwise, the patient was discharged without a HAPI; therefore, the label was negative, and the label time stamp was the discharge time.

The prediction time of 6 A.M. on the day of the label timestamp was chosen to provide a timely opportunity for clinical interventions, goals of care discussions, and resource planning.

**Figure 3:**
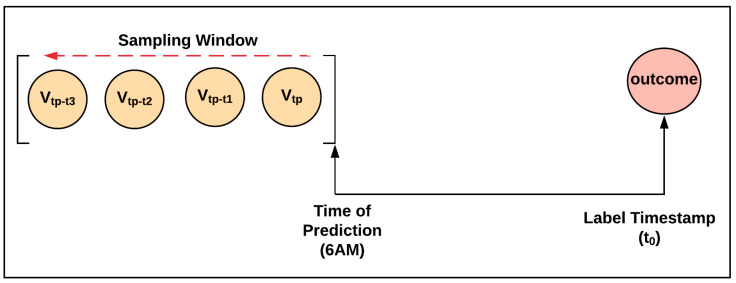
Sampling strategy for observational variables.

Clinical profiles were collected for each hospitalization, including vital signs, laboratory results, nursing assessments, and electrocardiograms. Time series were created for observational variables by looking backward from the prediction time (from tp), and the observational values were sampled at regular time intervals [18] depending on data availability (see Figure 3). Numerical variables with missing values were imputed with the median value [19] of the variable over the entire cohort at the sampling time point. Categorical values were normalized, and vectorized using one-hot encoding.

### 2.4. Clinical Feature Selection

The cohort was randomly split into a training set (7079/8855, 80%) and a test set (1776/8855, 20%), with no patient overlap between the sets. Because the HAPI rate was 18.6% (1648/8855) over the whole cohort, there was a class imbalance between the majority class (patients with no HAPI) and the minority class (patients with HAPIs). To address class imbalance and prevent the model from being biased toward the majority class, we applied an undersampling technique [20] to the training set. By reducing the number of majority-class samples, the model learns more effectively from minority-class examples, enhancing predictive performance for rare outcomes such as HAPIs.

An XGBoost [21] model was developed and optimized in Scala/Spark with the MLlib library [22] by using the train and test sets. XGBoost was selected for its interpretability, ability to handle imbalanced datasets, capture complex relationships, and efficiently process large-scale structured data with built-in regularization to prevent overfitting. Using ten-fold cross-validation integrated with a grid-search algorithm refined the hyperparameters based on the area under the receiver operating characteristic curve (AUROC). Grid search systematically explores different hyperparameter combinations to identify the optimal configuration, enhancing model performance and generalization. After the grid search, we implemented recursive feature elimination (RFE) [23]. RFE iteratively removes the least important features, improving model interpretability, reducing overfitting, and enhancing computational efficiency while maintaining predictive performance. This method involves removing one feature at a time and evaluating the model on the test set. We used AUROC to assess the model’s fit to the data, eliminating features that did not significantly impact the AUROC. The final optimal hyperparameters are listed in Appendix A Table A1. The final feature vector included 35 variables (192 features).

### 2.5. Internal and External Multi-Center Validation Sets

To assess the generalizability and robustness of our model, we validated it using both internal and external multi-center datasets that were not used during training and development. Initially, the model was developed and internally validated on patient data from Facility A, ensuring reliable performance within the institution. External validation, conducted on data from facilities B, C, D, and E, further tested the model’s robustness by exposing it to previously unseen populations.

The internal validation cohort included 1820 hospitalizations admitted to the same academic hospital (Facility A) from 1 January 2016 to 31 December 2017, as well as hospitalizations admitted from 16 January 2023 to 12 February 2023. To assess external generalizability, we included 1400 hospitalizations from Facility B, 839 hospitalizations from Facility C, 748 hospitalizations from Facility D, and 703 hospitalizations from Facility E. All 4 external cohorts consisted of admissions between 13 July 2021, and 17 March 2023.

This independent validation rigorously evaluates the model’s capacity to generalize beyond its development environment, providing a comprehensive assessment of its applicability across diverse clinical settings. This approach enhances the model’s potential for broader adoption, ensuring consistent and reliable performance in varied healthcare environments.

### 2.6. Benchmark Model: Braden Scale

The Braden Scale [24] was chosen as the benchmark to compare model performance given its widespread use as a pressure injury predictor in nursing. In all the Mount Sinai Health System facilities, the Braden Scale is used by registered nurses on every patient at every shift. The last Braden evaluation value before the label timestamp was kept for each patient, with a widely used cutoff value of under 12 predicting pressure injury risk.

### 2.7. Model Testing and Statistical Methods

For each of the developed models, performance was evaluated on the test set, the internal validation set (which was not used for model development) and the external validation sets (which were not used for model development either). The model-derived class probabilities were used to predict HAPI within 24 h. The final prediction threshold is selected such that there is a balance between sensitivity and specificity. Predictions less than the threshold were categorized as negative. Sensitivity, specificity, accuracy, positive predictive value, negative predictive value, F1-score, and AUROC, along with bootstrap 95% CIs, were estimated for evaluating the screening tool’s performance. Group comparisons were performed using a 2-sided Student *t* test or Kruskal–Wallis for continuous variables as appropriate and chi-square test for categorical variables. All analyses were performed using SciPy in Python 3.8.

## 3. Results

### 3.1. Study Population and Outcomes

A total of 8855 patients were included in the overall study cohort; clinical characteristics and demographics are summarized in Table 1. A total of 7079 patients were in the train set, and 1776 were in the test set. Moreover, 54.4% of the overall cohort was male, and the median age was 67.9 years old. The median length of stay was 12.3 days and ranged between 0.1 and 177 days. The overall rate of HAPI was 18.6% in the whole development cohort. There were significant statistical differences between patients with a HAPI and patients who were discharged without a HAPI for all key characteristics, except for age and most comorbidities. Notably, patients with a HAPI stayed hospitalized significantly longer, were more males, and had significantly more obesity than patients who were discharged without a HAPI (*p* < 0.001).

The internal validation set (facility A) included 1820 hospitalizations. The external validation sets from facilities B, C, D, and E included 1400, 839, 748, and 703 hospitalizations, respectively. The overall HAPI rates for the internal validation cohort at Facility A; and external validation cohorts at Facilities B, C, D, and E were 10.8%, 2.2%, 7.0%, 6.7%, and 5.0% The demographics and characteristics of each validation cohort are summarized in Table 2.

### 3.2. Labeling Logic Validation Results

**Figure 4:**
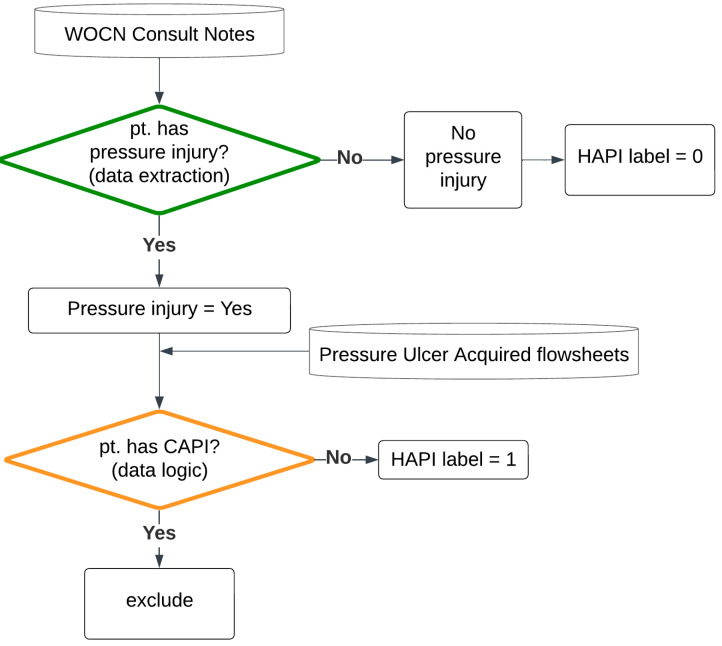
Data flow for the HAPI labeling logic.

The final validated labeling logic involves reading from various data sources: wound care consult notes and nursing assessment flowsheets for pressure ulcers. The logic data flow is described in the flow chart in Figure 4. The chart reviews resulted in an accuracy of 98% for that logic.

### 3.3. Predictors in the HAPI Predictive Model

**Figure 5:**
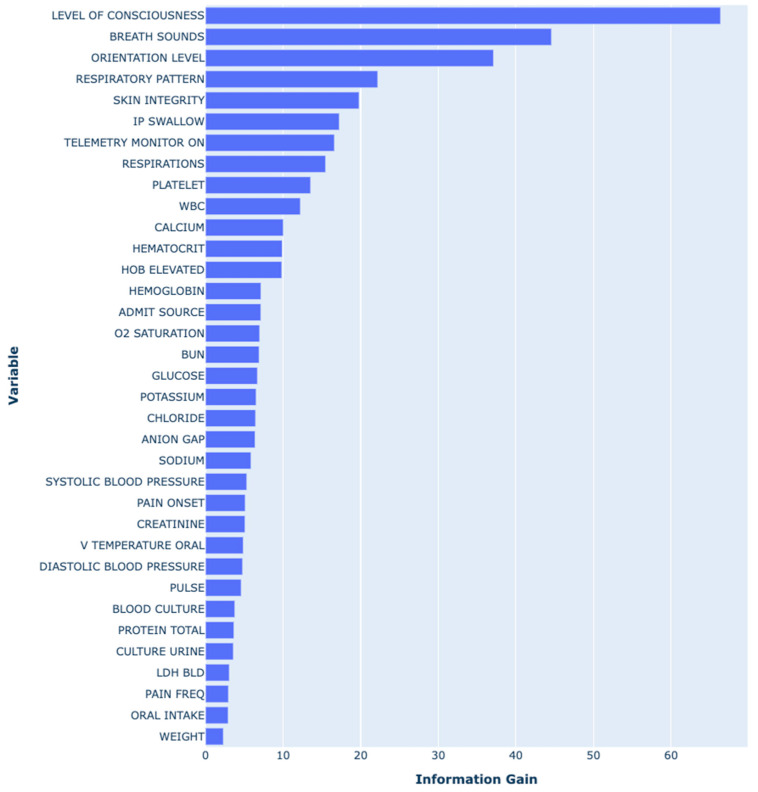
Variable importance of the final XGBoost model by descending information gain. Refer to Appendix A Table A2 for the definitions of the variables.

The final model was a Gradient Boosting model (XGBoost). Hyperparameters used in the final model are shown in Appendix A Table A1. Figure 5 summarizes the top predictive variables ordered by the Information Gain (the definitions of the variables in this figure are shown in Appendix A Table A2). Our model identified a series of features related to the level of activity (level of consciousness assessment and orientation-level assessment), the skin condition (skin integrity assessment), and respiratory status (breath sounds and respiratory pattern assessment). The level of consciousness assessment (the earliest recorded value of the latest three assessments) had the highest predictive value in the gradient boosting model, followed by breath sounds, orientation level, respiratory patterns, and skin integrity. The variables incorporated in the best model underscored the significance of temporal dynamics in patient mobility, respiratory function, and skin integrity.

### 3.4. Predictive Performance of the XGBoost Model

**Figure 6:**
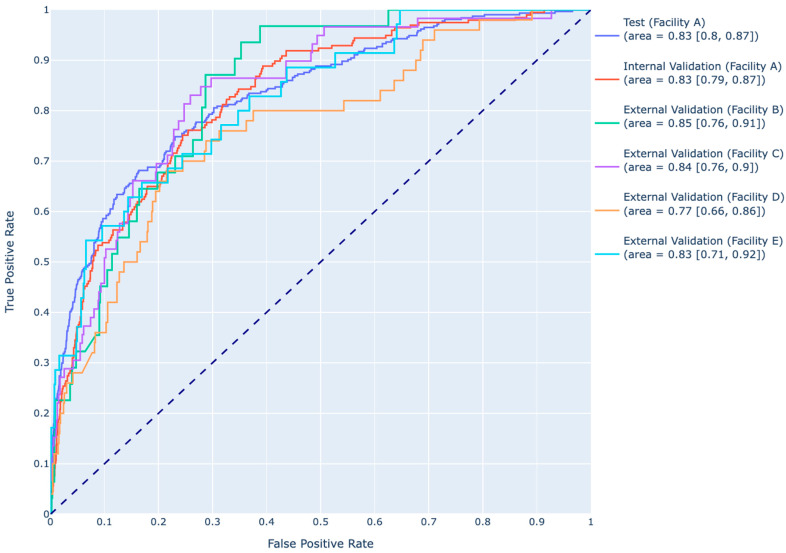
Receiver operating characteristic curves of the XGBoost model on the test set, the internal validation set, and all the external validation set, and their respective areas under the curve and 95% CIs.

At a prediction probability threshold of 0.48, for Facility A, the AUROC for the XGBoost model was 0.83 (95% CI: 0.80, 0.87) on the test set and 0.83 (95% CI: 0.79, 0.87) on the internal validation. For the external validation cohorts, the model gave an AUROC of 0.85 (95% CI: 0.76, 0.91) for Facility B, 0.84 (95% CI: 0.76, 0.90) for Facility C, 0.77 (95% CI: 0.66, 0.86) for Facility D, and 0.83 (95% CI: 0.71, 0.92) for Facility E. Figure 6 shows the receiver operating curves (ROCs) of the model test set, the internal validation set, and all external validation sets. Table 3 shows all the performance metrics for all the models on the test set, the internal validation set, and all external validation sets.

### 3.5. Comparison of the Predictive Performance of the Model to the Braden Scale Benchmark

**Figure 7:**
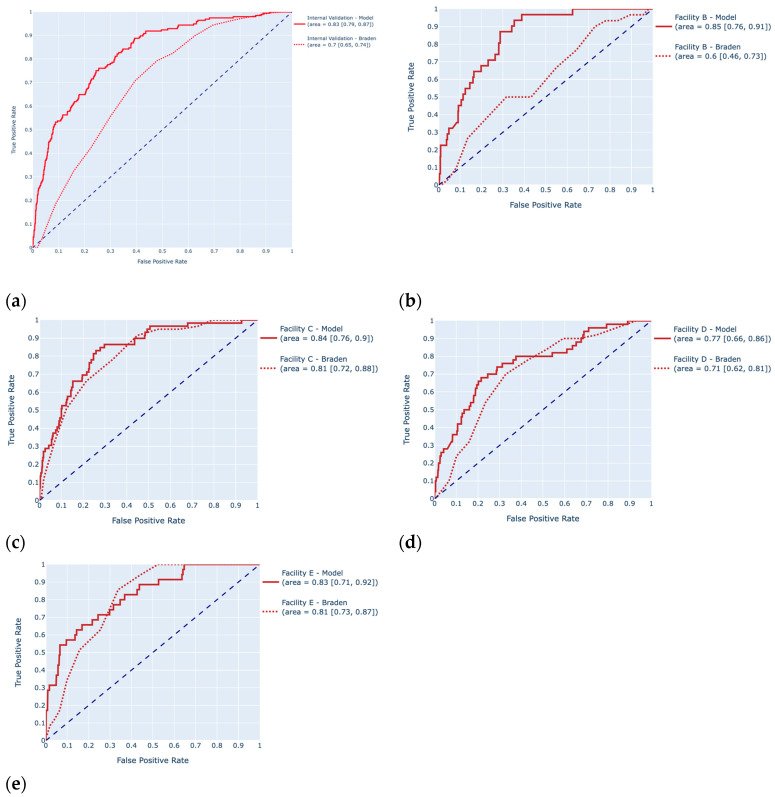
Comparison graphs of the respective receiver operating characteristic curves of the XGBoost model (solid lines) and the Braden Scale (dashed lines) on the internal validation set (**a**) and on the external validation sets (Facility B (**b**), Facility C (**c**), Facility D (**d**), Facility E (**e**)), and their respective areas under the curve and 95% CIs.

Compared to the Braden Scale, the model provided boosted performance results in sensitivity, AUROC and F1-score for the test set, the internal validation set, and all external validation sets. The sensitivity increased by more than 50%, from 50% to 76%, AUROC by 19%, F1-score by 34% on the test set. The F1-score increased by 50% on the internal validation set, by 129% on the Facility B set, by 10% on the Facility C set, 17% on the Facility D set, and by 35% on the Facility E set. The graphs comparing the ROC of the Braden Scale with the ROC of the model are shown in Figure 7.

## 4. Discussion

In this study, we developed and validated a machine learning application utilizing the XGBoost algorithm to predict the risk of HAPI. The application ingests raw data streams from EMR, including laboratory results, vital signs, and semi-structured clinical evaluations, to perform automated clinical profiling and generate HAPI risk scores. The integration of this application within the EMR ecosystem not only streamlines the clinical profiling process but also facilitates the implementation of machine learning (ML)-based screening process across the health system. Our approach demonstrated superior predictive performance compared to the widely used Braden Scale, surpassing it in terms of area under the receiver operating characteristic curve (AUROC), F1-score, sensitivity, and specificity across both internal and external validation cohorts.

### 4.1. Labeling Logic and Data Integrity

The availability of high-quality, curated datasets poses a significant challenge in developing ML models for clinical applications. Manual chart review, while effective, is resource-intensive, time-consuming, and financially prohibitive, especially when large-scale datasets are needed for robust model training and continuous updates.

To overcome these challenges, we developed an automated labeling pipeline using heuristic logic derived from the clinical expertise of wound care nurses (WOCNs). This pipeline utilized structured data from nursing assessments and consult notes within the EMR to identify HAPIs and accurately timestamp their onset. Validation through manual chart review demonstrated a labeling accuracy of 98%.

The automated pipeline not only ensured the creation of a reliable dataset for model training but also eliminated the inefficiencies and costs associated with manual methods. Furthermore, its integration within the EMR enables continuous learning and dynamic updates to the ML model as new data become available. This approach highlights the potential of automated labeling systems to advance scalable and cost-efficient development of ML models in healthcare, ensuring their adaptability to evolving clinical environments.

### 4.2. Incorporation of Temporal Data and Feature Selection

For clinical profiling, the majority of variables are observational in nature, representing repeated measurements where both the number of observations and the intervals between them vary across patients. A review of published models reveals that to address these inconsistencies, most approaches either exclude temporal data entirely or reformat input variables into fixed time-series windows. In these cases, data for longer stays are truncated, and shorter stays are padded with zeros to maintain a consistent input length [5,11,25,26,27,28,29].

In contrast, our approach employs a dynamic sampling module that looks backward from the prediction time, sampling observational values at regular intervals. This method allows for flexible sampling intervals, preserving temporal resolution and capturing trends that are more relevant to the prediction context. While fixed-window approaches are easier to implement and preprocess, they are less effective at capturing the variability in temporal dynamics across patients and clinical scenarios. Additionally, fixed-window methods may introduce bias by overemphasizing earlier data or underrepresenting critical recent observations.

Our backward-looking sampling approach provides significant advantages. It reduces potential bias by prioritizing recent data, improving the model’s ability to capture relevant temporal patterns and enhancing predictive performance. Moreover, this method is more generalizable, accommodating variability in patient trajectories by tailoring the sampling process to align with the prediction context. By emphasizing recent observations, it aligns with clinical reasoning and improves interpretability, supporting more informed and actionable decision-making.

We further optimized our model using recursive feature elimination (RFE), which systematically removed irrelevant features, resulting in a refined set of 35 key variables. This process not only improved the model’s predictive accuracy by reducing noise but also enhanced interpretability, making the clinical insights more actionable. Additionally, by focusing on the most impactful variables, RFE helped reduce the risk of overfitting, leading to better generalization on unseen data.

### 4.3. Key Predictors and Model Performance

The top predictive features in our model aligned with clinical understanding of HAPI risk factors, including level of consciousness, breath sounds, orientation, respiratory patterns, and skin integrity.

The majority of published predictive models heavily rely on length of stay (LOS) as a key predictor, which introduces significant limitations [11].

While LOS may serve as a proxy for risk, it is not causative; longer hospital stays inherently increase the opportunity for complications to occur but do not provide actionable insights into mitigating risk. Furthermore, LOS can be directly influenced by the occurrence of HAPIs, creating a circular dependency that biases model performance. This feedback loop challenges the validity of LOS as an independent predictor, as it may function simultaneously as both a predictor and an outcome of HAPIs. Additionally, patients with shorter hospital stays may remain at high risk for HAPIs, yet models heavily reliant on LOS may underestimate their risk. Hospitals with shorter average LOS may also experience reduced predictive performance when using such models.

In contrast, our model does not include LOS among the top 35 predictors (refer to the most important features figure). Instead, it prioritizes physiological and clinical indicators, focusing on variables directly associated with patient health and risk factors. This approach avoids the limitations associated with LOS dependency, allowing for a more robust and generalizable risk stratification across diverse clinical settings.

### 4.4. Practical Implications

The findings of this study have significant practical implications for quality of care and the prevention of hospital-acquired pressure injuries (HAPIs). The machine learning model we developed, with its strong predictive capabilities, offers a potential tool for real-time decision support within hospital settings. By integrating this XGBoost-based model into existing EHR systems, clinicians could be alerted about patients at high risk for HAPIs, allowing for early interventions such as more frequent repositioning, skin assessments, and the additional preventive measures.

Manual dataset labeling through chart review is costly, making machine learning model development an expensive endeavor. Our finding of an automated labeling approach, along with the methodology we used for label creation, could provide valuable insights for future researchers, enabling them to adopt and implement similar methods in their own research study.

Another important practical implication of our research is the technique we used to create features from clinical measurements. For routine measurements, we employed time-series data by sampling at regular intervals, while for non-routine clinical measurements, we used the last recorded value. This approach yielded superior results, outperforming the existing baseline model (Braden Scale). These findings could be valuable for feature engineering in clinical settings.

Overall, this model represents a step forward in leveraging machine learning in improving the quality of care and reducing adverse events in clinical care.

### 4.5. Limitations and Future Directions

While the model demonstrated consistent performance across multiple facilities within the Mount Sinai Health System in New York City, its generalizability may be constrained by the geographic and institutional scope of the study. All data used for model development and validation were sourced exclusively from hospitals within a single health system in New York City. This localized data context may not be representative of other geographic regions or healthcare systems, particularly those with differing patient populations, standards of care, or documentation practices. Future research should emphasize external validation in diverse healthcare settings, including non-academic hospitals, where variations in clinical workflows and documentation standards may affect model performance.

Additionally, the model was trained using labels generated by wound care specialists with specialized training and expertise in pressure injury assessment. However, the availability of certified WOCNs with such expertise is often limited. Following implementation, the primary users of the application are expected to be registered nurses (RNs), whose assessment practices may differ from those of WOCNs. Variability in agreement levels between RNs and the application could pose challenges for adoption and effective use, potentially impacting change management and the overall success of implementation. Addressing these disparities through training, feedback mechanisms, and iterative model refinements will be critical to ensuring effective integration and sustained clinical impact.

## 5. Conclusions

In this study, we developed and validated a machine learning model using XGBoost to predict HAPIs, outperforming the Braden Scale across multiple hospitals. By leveraging temporal clinical data and refining feature selection, and by using external validation cohorts from four different hospitals, the model demonstrated strong predictive accuracy and generalizability. While promising for real-time use in preventing HAPIs, further validation in varied health system environments is needed. Future research should prioritize enhancing the model’s prognostic value through the integration of multimodal data. This could include leveraging unstructured clinical data, such as progress notes in time-series formats, and incorporating medical imaging modalities to capture additional relevant features. Such advancements have the potential to further improve the model’s predictive performance and its utility in varied clinical environments.

## Figures and Tables

**Table 1:** Clinical characteristics and demographics of the development cohort.

Development Cohort (Facility A)			Overall	HAPI	No HAPI	*p*-Value
Admission	Number of hospitalizations		8855	1648	7207	
	LOS	Mean (SD)	17.7 (17.8)	19.4 (17.4)	17.3 (17.9)	<0.001
		Median [Min, Max]	12.3 [0.1, 177]	14.8 [0.1, 130]	11.5 [1, 177]	
Demographics	Age	Mean (SD)	66.4 (16.3)	67.0 (15.3)	66.3 (16.5)	0.12
		Median [Min, Max]	67.9 [18, 106.8]	68.1 [18, 103]	67.8 [18, 106.8]	
	Gender	Male	4823 (54.4%)	980 (59.5%)	3843 (53.3%)	<0.001
		Female	4007 (45.3%)	666 (40.4%)	3341 (46.4%)	
		Other	25 (0.3%)	2 (0.1%)	23 (0.3%)	
	Race and Ethnicity	White	2174 (24.6%)	472 (28.6%)	1702 (23.6%)	<0.001
		African American	1299 (14.7%)	256 (15.5%)	1043 (14.5%)	
		Hispanic	1938 (21.9%)	363 (22.0%)	1575 (21.9%)	
		Asian	337 (3.8%)	91 (5.5%)	246 (3.4%)	
		Other	2780 (31.4%)	388 (23.5%)	2392 (33.2%)	
		Unspecified	327 (3.7%)	78 (4.7%)	249 (3.5%)	
	BMI	Mean (SD)	27.4 (9.1)	26.1 (7.1)	27.8 (9.4)	<0.001
		Median [Min, Max]	25.9 [8.4, 240.7]	25.1 [8.4, 89.1]	26.1 [9.3, 240.7]	
Comorbidities	Elixhauser Score	Mean (SD)	23.4 (19.9)	30.5 (19.8)	21.5 (19.6)	<0.001
		Median [Min, Max]	21 [−33, 106]	29 [−24, 92]	20 [−33, 106]	
	Diabetes	Yes	3990 (45.1%)	740 (44.9%)	3250 (45.1%)	0.17
		No	4802 (54.2%)	902 (54.7%)	3900 (54.1%)	
		Missing	63 (0.7%)	6 (0.4%)	57 (0.8%)	
	Obesity	Yes	1850 (20.9%)	286 (17.3%)	1564 (21.7%)	<0.001
		No	6942 (78.4%)	1356 (82.3%)	5586 (77.5%)	
		Missing	63 (0.7%)	6 (0.4%)	57 (0.8%)	
	Dementia	Yes	887 (10.0%)	174 (10.5%)	713 (9.9%)	0.13
		No	7905 (89.3%)	1468 (89.1%)	6437 (89.3%)	
		Missing	63 (0.7%)	6 (0.4%)	57 (0.8%)	
	Renal Failure	Yes	3301 (37.3%)	637 (38.6%)	2664 (37.0%)	0.09
		No	5491 (62.0%)	1005 (61.0%)	4486 (62.2%)	
		Missing	63 (0.7%)	6 (0.4%)	57 (0.8%)	
	Heart Failure	Yes	3334 (37.7%)	643 (39.0%)	2691 (37.3%)	0.09
		No	5458 (61.6%)	999 (60.6%)	4459 (61.9%)	
		Missing	63 (0.7%)	6 (0.4%)	57 (0.8%)	
	Liver Disease	Yes	1615 (18.2%)	331 (20.1%)	1284 (17.8%)	0.02
		No	7177 (81.1%)	1311 (79.5%)	5866 (81.4%)	
		Missing	63 (0.7%)	6 (0.4%)	57 (0.8%)	
	Autoimmune disease	Yes	460 (5.2%)	72 (4.3%)	388 (5.4%)	0.05
		No	8332 (94.1%)	1570 (95.3%)	6762 (93.8%)	
		Missing	63 (0.7%)	6 (0.4%)	57 (0.8%)	
Braden Scale		Mean (SD)	15.2 (3.9)	12.9 (3.0)	15.7 [3.9]	<0.001
		Median [Min, Max]	15 [6, 23]	12 [6, 22]	16 [6, 23]	
Hospital-Acquired Pressure Injury Outcome			1648 (18.6%)			

**Table 2:** Clinical characteristics and demographics of the validation cohorts.

Validation Cohorts			Internal Validation	External Validation			
			Facility A	Facility B	Facility C	Facility D	Facility E
Admission	Number of hospitalizations		1820	1400	839	748	703
	LOS	Mean (SD)	48.6 (16.6)	16.7 (16.4)	14.2 (12.3)	20.5 (22.3)	12.9 (12.7)
		Median [Min, Max]	12 [2, 176]	12 [2, 149]	10 [2, 103]	13.4 [2, 164]	8.6 [2, 105]
Demographics	Age	Mean (SD)	65.6 (17.5)	73.4 (15.3)	75.1 (15.0)	70.7 (15.8)	69.4 (16.5)
		Median [Min, Max]	67.2 [18, 109]	75.8 [18, 107]	76.8 [22, 106]	72.5 [18, 105]	70.1 [23.7, 122]
	Gender	Male	991 (54.4%)	664 (47.4)	390 (46.5%)	328 (43.8%)	415 (59.0%)
		Female	828 (45.5%)	719 (51.4%)	445 (53.0%)	417 (55.8%)	284 (40.4%)
		Other	1 (0.1%)	17 (1.2%)	4 (0.5%)	3 (0.4%)	4 (0.6%)
	Race and Ethnicity	White	491 (27.0%)	203 (14.5%)	303 (36.1%)	271 (36.2%)	156 (22.2%)
		African American	271 (14.9%)	328 (23.4%)	217 (25.9%)	111 (14.8%)	102 (14.5%)
		Hispanic	190 (10.4%)	337 (24.1%)	28 (3.3%)	125 (16.7%)	177 (25.2%)
		Asian	41 (2.3%)	27 (1.9%)	21 (2.5%)	32 (4.3%)	45 (6.4%)
		Other	742 (40.8%)	473 (33.8%)	254 (30.3%)	198 (26.5%)	189 (26.9%)
		Unspecified	85 (4.7%)	32 (2.3%)	16 (1.9%)	11 (1.5%)	34 (4.8%)
	BMI	Mean (SD)	27.9 (9.9)	26.2 (9.3)	28.3 (9.1)	26.8 (12.0)	26.9 (13.1)
		Median [Min, Max]	26.0 [9.5, 181.8]	24.3 [11.8, 154.7]	26.5 [12.3, 76.1]	25.1 [11.8, 277.4]	24.7 [11.9, 281.2]
Comorbidities	Elixhauser Score	Mean (SD)	19.1 (19.0)	22.7 (20.0)	18.3 (19.5)	22.5 (20.8)	17.4 (19.9)
		Median [Min, Max]	17 [−24, 95]	20 [−26, 105]	16 [−30, 85]	22 [−23, 95]	16 [−30, 88]
	Diabetes	Yes	821 (45.1%)	619 (44.2%)	399 (47.6%)	239 (32.0%)	294 (41.8%)
		No	978 (53.7%)	749 (53.5%)	399 (47.5%)	479 (64.0%)	374 (53.2%)
		Missing	21 (1.2%)	32 (2.3%)	41 (4.9%)	30 (4.0%)	35 (5.0%)
	Obesity	Yes	396 (21.7%)	225 (16.1%)	233 (27.8%)	114 (15.2%)	133 (18.9%)
		No	1403 (77.1%)	1143 (81.6%)	565 (67.3%)	604 (20.8%)	535 (76.1%)
		Missing	21 (1.2%)	32 (2.3%)	41 (4.9%)	30 (4.0%)	35 (5.0%)
	Dementia	Yes	199 (10.9%)	335 (23.9%)	205 (24.4%)	84 (11.2%)	105 (14.9%)
		No	1600 (87.9%)	1033 (73.8%)	593 (70.7%)	634 (84.8%)	563 (80.1%)
		Missing	21 (1.2%)	32 (2.3%)	41 (4.9%)	30 (4.0%)	35 (5.0%)
	Renal Failure	Yes	699 (38.4%)	457 (32.6%)	263 (31.3%)	188 (25.1%)	225 (32.0%)
		No	1100 (60.4%)	911 (65.1%)	535 (63.8%)	530 (70.9%)	443 (63.0%)
		Missing	21 (1.2%)	32 (2.3%)	41 (4.9%)	30 (4.0%)	35 (5.0%)
	Heart Failure	Yes	593 (32.6%)	505 (36.1%)	277 (33.0%)	212 (28.3%)	226 (32.1%)
		No	1206 (66.2%)	863 (61.6%)	521 (62.1%)	506 (37.7%)	442 (62.9%)
		Missing	21 (1.2%)	32 (2.3%)	41 (4.9%)	30 (4.0%)	35 (5.0%)
	Liver Disease	Yes	320 (17.6%)	112 (8.0%)	70 (8.3%)	78 (10.4%)	89 (12.7%)
		No	1479 (81.2%)	1256 (89.7%)	728 (86.8%)	640 (85.6%)	579 (82.3%)
		Missing	21 (1.2%)	32 (2.3%)	41 (4.9%)	30 (4.0%)	35 (5.0%)
	Autoimmune disease	Yes	93 (5.1%)	63 (4.5%)	20 (2.4%)	26 (3.5%)	35 (5.0%)
		No	1706 (93.7%)	1305 (93.2%)	778 (92.7%)	692 (92.5%)	633 (90.5%)
		Missing	21 (1.2%)	32 (2.3%)	41 (4.9%)	30 (4.0%)	35 (5.0%)
Braden Scale		Mean (SD)	15.4 (3.8)	14.4 [3.6]	15.0 (3.4)	15.2 (3.7)	15.9 (3.5)
		Median [Min, Max]	15 [6, 23]	14 [6, 23]	15 [6, 23]	15 [6, 23]	16 [3, 23]
Hospital-Acquired Pressure Injury Outcome			197 (10.8%)	31 (2.2%)	59 (7.0%)	50 (6.7%)	35 (5.0%)

**Table 3:** Predictive performance of both Braden Scale and the XGBoost model on the test set (Facility A), the internal validation set (Facility A), and the external validation sets (Facilities B, C, D, E). Positive and negative predictions were assigned using the prediction probability threshold of 0.48.

Dataset	Model	Accuracy (95% CI)	Sensitivity (95% CI)	Specificity (95% CI)	Precision (95% CI)	AUROC (95% CI)	F1-Score (95% CI)
Test (Facility A)	Braden Scale	0.72 (0.68, 0.74)	0.50 (0.42, 0.57)	0.76 [0.73, 0.80)	0.31 (0.26, 0.36)	0.70 (0.66, 0.74)	0.38 (0.32, 0.44)
	HAPI model	0.74 (0.71, 0.77)	0.76 (0.69, 0.82)	0.74 (0.71, 0.77)	0.39 (0.33, 0.44)	0.83 (0.79, 0.87)	0.51 (0.46, 0.56)
Internal Validation (Facility A)	Braden Scale	0.74 (0.71, 0.77)	0.42 (0.33, 0.52)	0.78 (0.75, 0.81)	0.18 (0.14, 0.23)	0.70 (0.65, 0.74)	0.26 (0.19, 0.32)
	HAPI model	0.75 (0.73, 0.78)	0.74 (0.65, 0.83)	0.76 (0.73, 0.79)	0.27 (0.22, 0.32)	0.83 (0.90, 0.87)	0.39 (0.33, 0.45)
External Validation (Facility B)	Braden Scale	0.68 (0.65, 0.72)	0.5 (0.25, 0.73)	0.68 (0.65, 0.72)	0.03 (0.01, 0.06)	0.61 (0.47, 0.75)	0.07 (0.03, 0.11)
	HAPI model	0.87 (0.84, 0.89)	0.54 (0.29, 0.79)	0.88 (0.85, 0.9)	0.09 (0.04, 0.15)	0.85 (0.76, 0.91)	0.16 (0.07, 0.25)
External Validation (Facility C)	Braden Scale	0.78 (0.74, 0.82)	0.65 (0.47, 0.82)	0.79 (0.74, 0.82)	0.19 (0.12, 0.27)	0.81 (0.73, 0.87)	0.30 (0.20, 0.40)
	HAPI model	0.90 (0.86, 0.92)	0.38 (0.19, 0.54)	0.93 (0.90, 0.96)	0.29 (0.16, 0.46)	0.84 (0.76, 0.90)	0.33 (0.18, 0.47)
External Validation (Facility D)	Braden Scale	0.75 (0.71, 0.79)	0.54 (0.35, 0.74)	0.77 (0.72, 0.81)	0.14 (0.08, 0.22)	0.71 (0.61, 0.81)	0.23 (0.11, 0.32)
	HAPI model	0.85 (0.81, 0.88)	0.42 (0.24, 0.62)	0.88 (0.84, 0.91)	0.2 (0.10, 0.34)	0.77 (0.66, 0.86)	0.27 (0.14, 0.39)
External Validation (Facility E)	Braden Scale	0.83 (0.79, 0.87)	0.52 (0.28, 0.77)	0.84 (0.80, 0.88)	0.14 (0.06, 0.23)	0.81 (0.73, 0.88)	0.23 (0.10, 0.35)
	HAPI model	0.87 (0.84, 0.91)	0.56 (0.31, 0.82)	0.89 (0.85, 0.92)	0.21 (0.10, 0.34)	0.83 (0.71, 0.92)	0.31 (0.17, 0.44)

## Data Availability

The raw data underlying this article were generated at the Mount Sinai Health System. Derived data supporting the findings may be available from the corresponding author (K.N.) upon request.

## Appendix A

**Table A1:** Hyperparameters used in the final XGBoost model.

Hyperparameter	Search Space	Optimal Value Used in the Final Model
eta	[0.005, 0.01, 0.05]	0.01
gamma	[0.5, 1, 5, 10]	0.5
max depth	[2, 3, 4, 5]	3
lambda	[0.5, 1, 5, 10]	10
alpha	[0.1, 0.05, 1, 5, 10]	0.1

**Table A2:** Variables included in the final XGBoost model and their respective data source.

Full Variable Name	Variable Name	Source of Data	Unit
Admission source	ADMIT SOURCE	Visit	
Temperature	V TEMPERATURE ORAL	Vitals Flowsheet	degree Fahrenheit
Diastolic blood pressure	DIASTOLIC BLOOD PRESSURE	Vitals Flowsheet	rate per minute
Systolic blood pressure	SYSTOLIC BLOOD PRESSURE	Vitals Flowsheet	mmHg
Percutaneous oxygen saturation	O2 SATURATION	Vitals Flowsheet	%
Pulse	PULSE	Vitals Flowsheet	rate per minute
Respiratory rate	RESPIRATIONS	Vitals Flowsheet	rate per minute
Weight	WEIGHT	Vitals Flowsheet	lb
Anion gap	ANION GAP	Laboratory	mEq/L
Blood culture	BLOOD CULTURE	Laboratory	
Blood urea nitrogen	BUN	Laboratory	mg/dL
Serum calcium	CALCIUM	Laboratory	mg/dL
Serum chloride	CHLORIDE	Laboratory	mmol/L
Urine culture	CULTURE_URINE	Laboratory	
Serum Creatinine	CREATININE	Laboratory	mg/dL
Blood glucose	GLUCOSE	Laboratory	mg/dL
Hematocrit	HEMATOCRIT	Laboratory	%
Hemoglobin	HEMOGLOBIN	Laboratory	g/dL
Lactate dehydrogenase	LDH BLD	Laboratory	U/L
Platelet count	PLATELET	Laboratory	×10^3^/μL
Serum potassium	POTASSIUM	Laboratory	mEq/L
Serum total protein	PROTEIN TOTAL	Laboratory	g/dL
Serum sodium	SODIUM	Laboratory	mEq/L
WBC count	WBC	Laboratory	×10^3^/μL
Breath sound assessment	BREATH SOUNDS	Nursing Assessment Flowsheet	
Head of bed elevation (angle)	HOB ELEVATED	Nursing Assessment Flowsheet	
Inpatient swallow screening	IP SWALLOW	Nursing Assessment Flowsheet	
Level of consciousness screening	LEVEL OF CONSCIOUSNESS	Nursing Assessment Flowsheet	
Oral intake	ORAL INTAKE	Nursing Assessment Flowsheet	
Orientation assessment	ORIENTATION LEVEL	Nursing Assessment Flowsheet	
Pain frequency assessment	PAIN FREQ	Nursing Assessment Flowsheet	
Pain onset	PAIN ONSET	Nursing Assessment Flowsheet	
Respiratory pattern assessment	RESPIRATORY PATTERN	Nursing Assessment Flowsheet	
Skin integrity assessment	SKIN INTEGRITY	Nursing Assessment Flowsheet	
Is the telemetry monitor on?	TELEMETRY MONITOR ON	Nursing Assessment Flowsheet	
